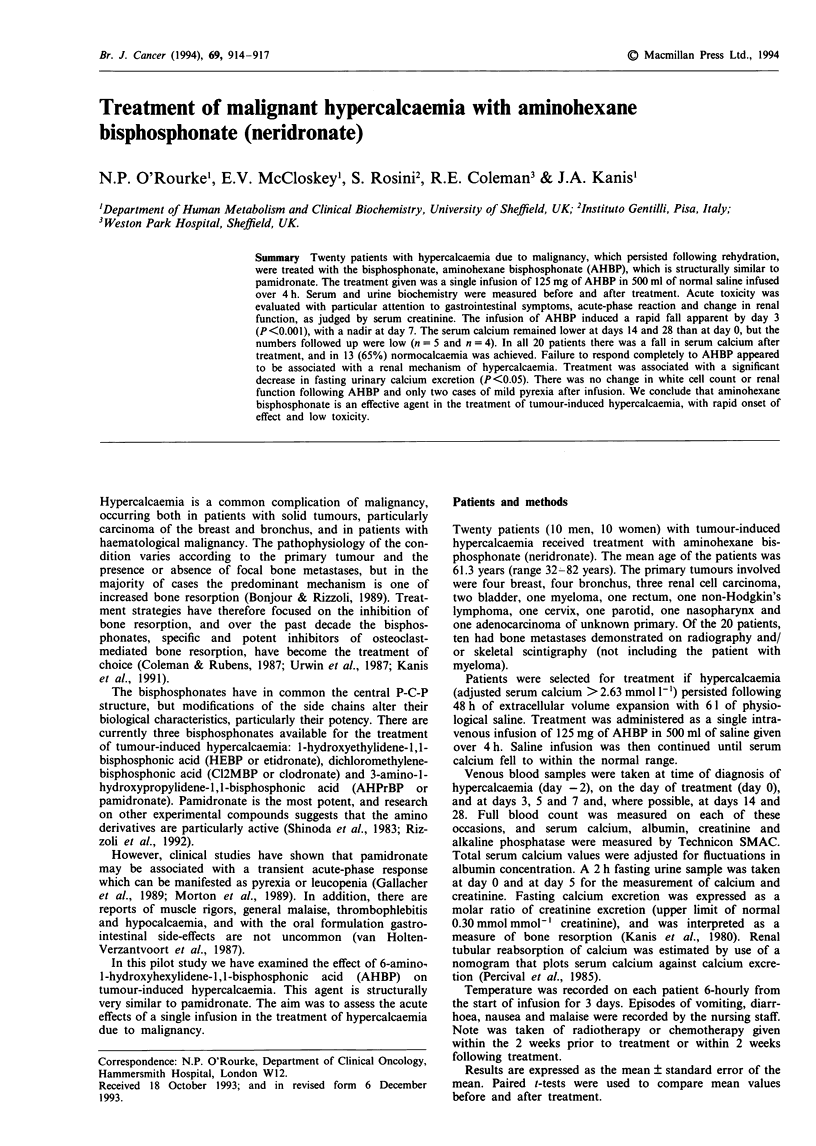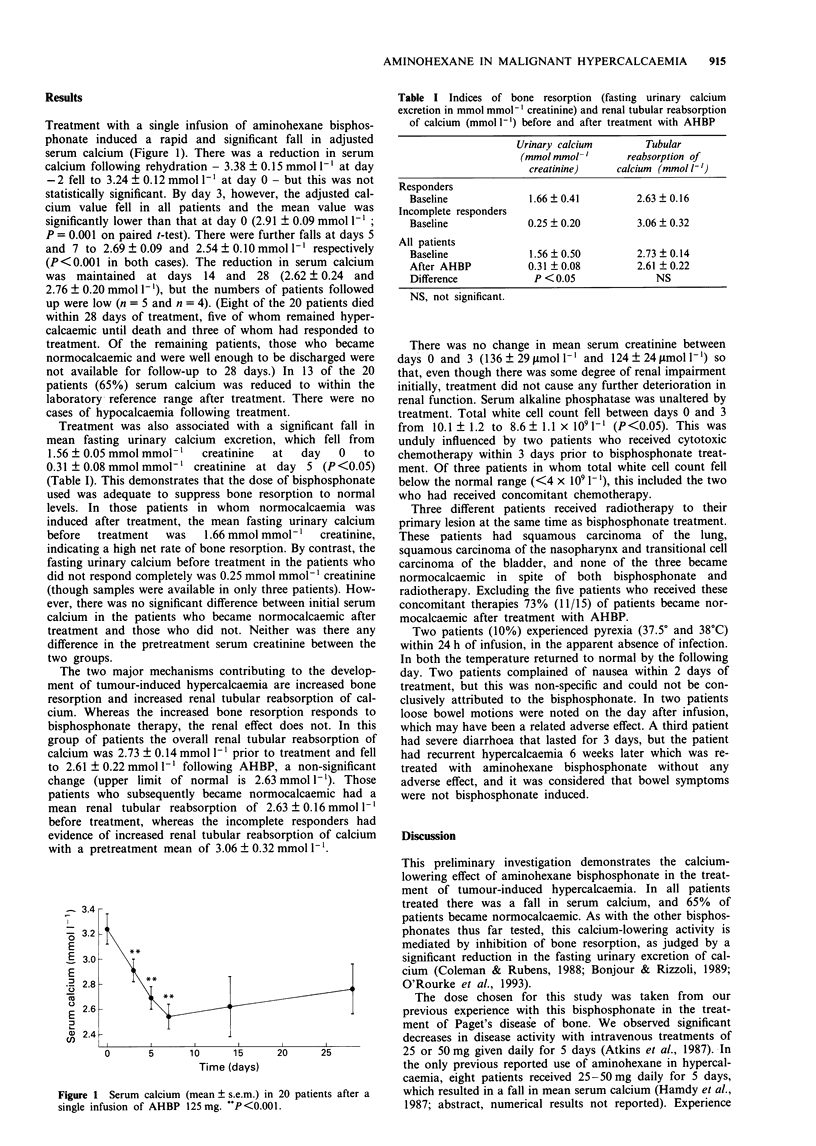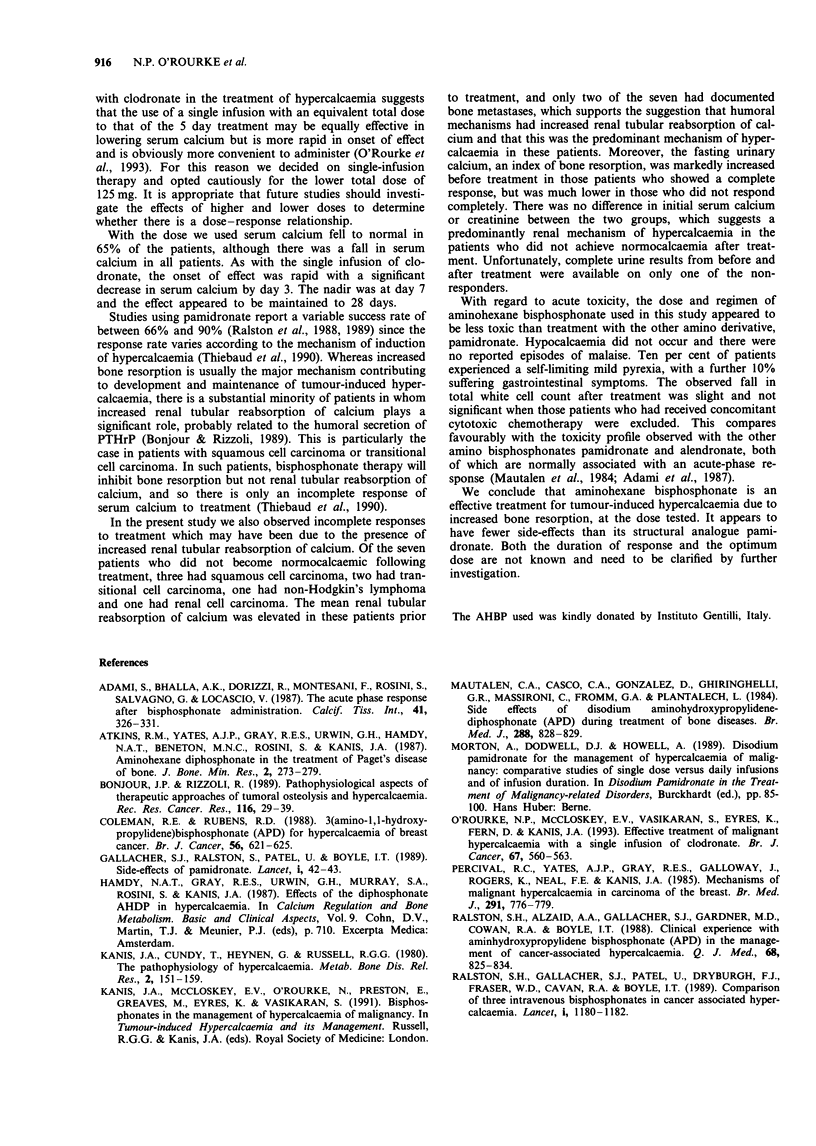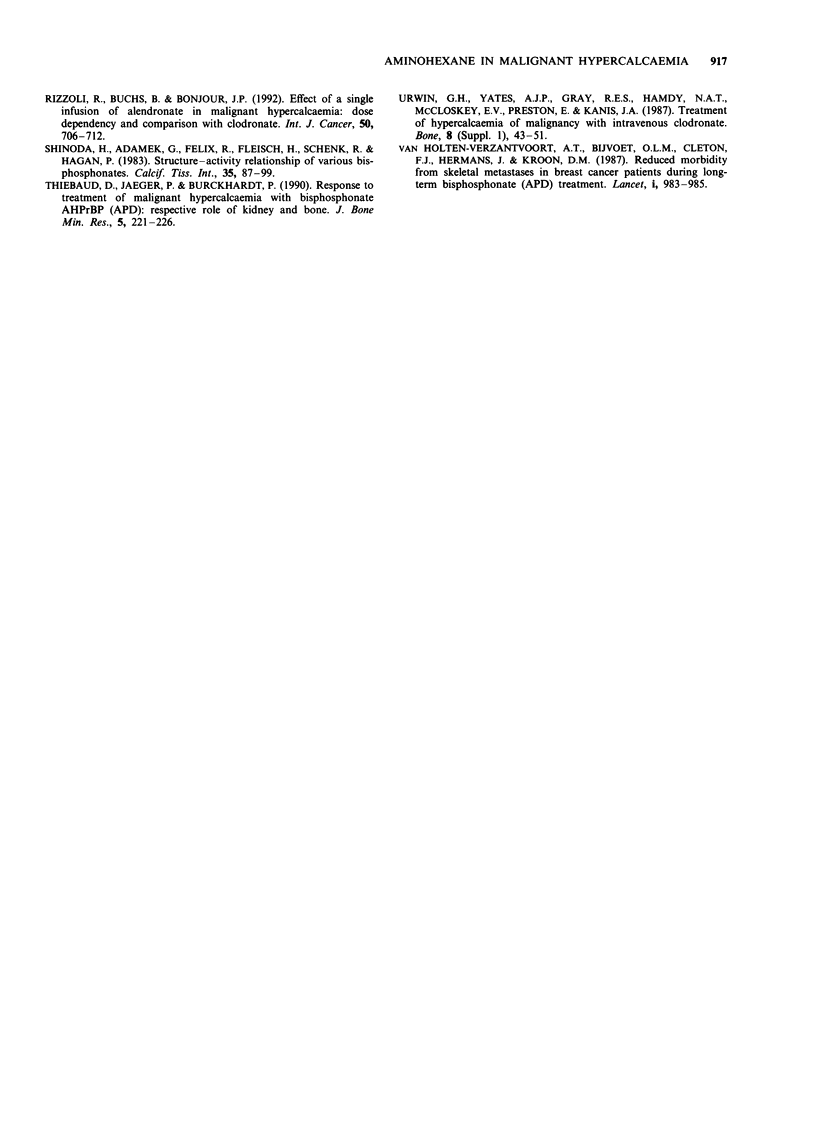# Treatment of malignant hypercalcaemia with aminohexane bisphosphonate (neridronate).

**DOI:** 10.1038/bjc.1994.176

**Published:** 1994-05

**Authors:** N. P. O'Rourke, E. V. McCloskey, S. Rosini, R. E. Coleman, J. A. Kanis

**Affiliations:** Department of Human Metabolism and Clinical Biochemistry, University of Sheffield, UK.

## Abstract

Twenty patients with hypercalcaemia due to malignancy, which persisted following rehydration, were treated with the bisphosphonate, aminohexane bisphosphonate (AHBP), which is structurally similar to pamidronate. The treatment given was a single infusion of 125 mg of AHBP in 500 ml of normal saline infused over 4 h. Serum and urine biochemistry were measured before and after treatment. Acute toxicity was evaluated with particular attention to gastrointestinal symptoms, acute-phase reaction and change in renal function, as judged by serum creatinine. The infusion of AHBP induced a rapid fall apparent by day 3 (P < 0.001), with a nadir at day 7. The serum calcium remained lower at days 14 and 28 than at day 0, but the numbers followed up were low (n = 5 and n = 4). In all 20 patients there was a fall in serum calcium after treatment, and in 13 (65%) normocalcaemia was achieved. Failure to respond completely to AHBP appeared to be associated with a renal mechanism of hypercalcaemia. Treatment was associated with a significant decrease in fasting urinary calcium excretion (P < 0.05). There was no change in white cell count or renal function following AHBP and only two cases of mild pyrexia after infusion. We conclude that aminohexane bisphosphonate is an effective agent in the treatment of tumour-induced hypercalcaemia, with rapid onset of effect and low toxicity.


Br. J. Cancer (1994), 69, 914-917 © Macmillan Press Ltd., 1994

### Treatment of malignant hypercalcaemia with aminohexane bisphosphonate (neridronate)

N.P. O'Rourke[1], E.V. McCloskey[1], S. Rosini[2], R.E. Coleman[3] & J.A. Kanis[1]

[1]*Department of Human Metabolism and Clinical Biochemistry, University of Sheffield, UK;* [2]*Instituto Gentilli, Pisa, Italy;* [3]*Weston Park Hospital, Sheffield, UK.*

**Summary** Twenty patients with hypercalcaemia due to malignancy, which persisted following rehydration, were treated with the bisphosphonate, aminohexane bisphosphonate (AHBP), which is structurally similar to pamidronate. The treatment given was a single infusion of 125 mg of AHBP in 500 ml of normal saline infused over 4 h. Serum and urine biochemistry were measured before and after treatment. Acute toxicity was evaluated with particular attention to gastrointestinal symptoms, acute-phase reaction and change in renal function, as judged by serum creatinine. The infusion of AHBP induced a rapid fall apparent by day 3 ($P<0.001$), with a nadir at day 7. The serum calcium remained lower at days 14 and 28 than at day 0, but the numbers followed up were low ($n = 5$ and $n = 4$). In all 20 patients there was a fall in serum calcium after treatment, and in 13 (65%) normocalcaemia was achieved. Failure to respond completely to AHBP appeared to be associated with a renal mechanism of hypercalcaemia. Treatment was associated with a significant decrease in fasting urinary calcium excretion ($P<0.05$). There was no change in white cell count or renal function following AHBP and only two cases of mild pyrexia after infusion. We conclude that aminohexane bisphosphonate is an effective agent in the treatment of tumour-induced hypercalcaemia, with rapid onset of effect and low toxicity.

Hypercalcaemia is a common complication of malignancy, occurring both in patients with solid tumours, particularly carcinoma of the breast and bronchus, and in patients with haematological malignancy. The pathophysiology of the condition varies according to the primary tumour and the presence or absence of focal bone metastases, but in the majority of cases the predominant mechanism is one of increased bone resorption (Bonjour & Rizzoli, 1989). Treatment strategies have therefore focused on the inhibition of bone resorption, and over the past decade the bisphosphonates, specific and potent inhibitors of osteoclast-mediated bone resorption, have become the treatment of choice (Coleman & Rubens, 1987; Urwin *et al.*, 1987; Kanis *et al.*, 1991).

The bisphosphonates have in common the central P-C-P structure, but modifications of the side chains alter their biological characteristics, particularly their potency. There are currently three bisphosphonates available for the treatment of tumour-induced hypercalcaemia: 1-hydroxyethylidene-1,1-bisphosphonic acid (HEBP or etidronate), dichloromethylene-bisphosphonic acid (Cl2MBP or clodronate) and 3-amino-1-hydroxypropylidene-1,1-bisphosphonic acid (AHPrBP or pamidronate). Pamidronate is the most potent, and research on other experimental compounds suggests that the amino derivatives are particularly active (Shinoda *et al.*, 1983; Rizzoli *et al.*, 1992).

However, clinical studies have shown that pamidronate may be associated with a transient acute-phase response which can be manifested as pyrexia or leucopenia (Gallacher *et al.*, 1989; Morton *et al.*, 1989). In addition, there are reports of muscle rigors, general malaise, thrombophlebitis and hypocalcaemia, and with the oral formulation gastrointestinal side-effects are not uncommon (van Holten-Verzantvoort *et al.*, 1987).

In this pilot study we have examined the effect of 6-amino-1-hydroxyhexylidene-1,1-bisphosphonic acid (AHBP) on tumour-induced hypercalcaemia. This agent is structurally very similar to pamidronate. The aim was to assess the acute effects of a single infusion in the treatment of hypercalcaemia due to malignancy.

Correspondence: N.P. O'Rourke, Department of Clinical Oncology, Hammersmith Hospital, London W12.
Received 18 October 1993; and in revised form 6 December 1993.

### Patients and methods

Twenty patients (10 men, 10 women) with tumour-induced hypercalcaemia received treatment with aminohexane bisphosphonate (neridronate). The mean age of the patients was 61.3 years (range 32–82 years). The primary tumours involved were four breast, four bronchus, three renal cell carcinoma, two bladder, one myeloma, one rectum, one non-Hodgkin's lymphoma, one cervix, one parotid, one nasopharynx and one adenocarcinoma of unknown primary. Of the 20 patients, ten had bone metastases demonstrated on radiography and/or skeletal scintigraphy (not including the patient with myeloma).

Patients were selected for treatment if hypercalcaemia (adjusted serum calcium $>2.63\ mmol\ l^{-1}$) persisted following 48 h of extracellular volume expansion with 6 l of physiological saline. Treatment was administered as a single intravenous infusion of 125 mg of AHBP in 500 ml of saline given over 4 h. Saline infusion was then continued until serum calcium fell to within the normal range.

Venous blood samples were taken at time of diagnosis of hypercalcaemia (day −2), on the day of treatment (day 0), and at days 3, 5 and 7 and, where possible, at days 14 and 28. Full blood count was measured on each of these occasions, and serum calcium, albumin, creatinine and alkaline phosphatase were measured by Technicon SMAC. Total serum calcium values were adjusted for fluctuations in albumin concentration. A 2 h fasting urine sample was taken at day 0 and at day 5 for the measurement of calcium and creatinine. Fasting calcium excretion was expressed as a molar ratio of creatinine excretion (upper limit of normal $0.30\ mmol\ mmol^{-1}$ creatinine), and was interpreted as a measure of bone resorption (Kanis *et al.*, 1980). Renal tubular reabsorption of calcium was estimated by use of a nomogram that plots serum calcium against calcium excretion (Percival *et al.*, 1985).

Temperature was recorded on each patient 6-hourly from the start of infusion for 3 days. Episodes of vomiting, diarrhoea, nausea and malaise were recorded by the nursing staff. Note was taken of radiotherapy or chemotherapy given within the 2 weeks prior to treatment or within 2 weeks following treatment.

Results are expressed as the mean ± standard error of the mean. Paired *t*-tests were used to compare mean values before and after treatment.

### Results

Treatment with a single infusion of aminohexane bisphosphonate induced a rapid and significant fall in adjusted serum calcium (Figure 1). There was a reduction in serum calcium following rehydration – $3.38 \pm 0.15$ mmol $l^{-1}$ at day −2 fell to $3.24 \pm 0.12$ mmol $l^{-1}$ at day 0 – but this was not statistically significant. By day 3, however, the adjusted calcium value fell in all patients and the mean value was significantly lower than that at day 0 ($2.91 \pm 0.09$ mmol $l^{-1}$; $P = 0.001$ on paired $t$-test). There were further falls at days 5 and 7 to $2.69 \pm 0.09$ and $2.54 \pm 0.10$ mmol $l^{-1}$ respectively ($P<0.001$ in both cases). The reduction in serum calcium was maintained at days 14 and 28 ($2.62 \pm 0.24$ and $2.76 \pm 0.20$ mmol $l^{-1}$), but the numbers of patients followed up were low ($n = 5$ and $n = 4$). (Eight of the 20 patients died within 28 days of treatment, five of whom remained hypercalcaemic until death and three of whom had responded to treatment. Of the remaining patients, those who became normocalcaemic and were well enough to be discharged were not available for follow-up to 28 days.) In 13 of the 20 patients (65%) serum calcium was reduced to within the laboratory reference range after treatment. There were no cases of hypocalcaemia following treatment.

Treatment was also associated with a significant fall in mean fasting urinary calcium excretion, which fell from $1.56 \pm 0.05$ mmol $mmol^{-1}$ creatinine at day 0 to $0.31 \pm 0.08$ mmol $mmol^{-1}$ creatinine at day 5 ($P<0.05$) (Table I). This demonstrates that the dose of bisphosphonate used was adequate to suppress bone resorption to normal levels. In those patients in whom normocalcaemia was induced after treatment, the mean fasting urinary calcium before treatment was 1.66 mmol $mmol^{-1}$ creatinine, indicating a high net rate of bone resorption. By contrast, the fasting urinary calcium before treatment in the patients who did not respond completely was 0.25 mmol $mmol^{-1}$ creatinine (though samples were available in only three patients). However, there was no significant difference between initial serum calcium in the patients who became normocalcaemic after treatment and those who did not. Neither was there any difference in the pretreatment serum creatinine between the two groups.

The two major mechanisms contributing to the development of tumour-induced hypercalcaemia are increased bone resorption and increased renal tubular reabsorption of calcium. Whereas the increased bone resorption responds to bisphosphonate therapy, the renal effect does not. In this group of patients the overall renal tubular reabsorption of calcium was $2.73 \pm 0.14$ mmol $l^{-1}$ prior to treatment and fell to $2.61 \pm 0.22$ mmol $l^{-1}$ following AHBP, a non-significant change (upper limit of normal is 2.63 mmol $l^{-1}$). Those patients who subsequently became normocalcaemic had a mean renal tubular reabsorption of $2.63 \pm 0.16$ mmol $l^{-1}$ before treatment, whereas the incomplete responders had evidence of increased renal tubular reabsorption of calcium with a pretreatment mean of $3.06 \pm 0.32$ mmol $l^{-1}$.

**Figure 1** Serum calcium (mean ± s.e.m.) in 20 patients after a single infusion of AHBP 125 mg. $^{**}P<0.001$.

**Table I** Indices of bone resorption (fasting urinary calcium excretion in mmol $mmol^{-1}$ creatinine) and renal tubular reabsorption of calcium (mmol $l^{-1}$) before and after treatment with AHBP

| | *Urinary calcium (mmol mmol⁻¹ creatinine)* | *Tubular reabsorption of calcium (mmol l⁻¹)* |
|---|---|---|
| Responders | | |
| Baseline | $1.66 \pm 0.41$ | $2.63 \pm 0.16$ |
| Incomplete responders | | |
| Baseline | $0.25 \pm 0.20$ | $3.06 \pm 0.32$ |
| All patients | | |
| Baseline | $1.56 \pm 0.50$ | $2.73 \pm 0.14$ |
| After AHBP | $0.31 \pm 0.08$ | $2.61 \pm 0.22$ |
| Difference | $P<0.05$ | NS |

NS, not significant.

There was no change in mean serum creatinine between days 0 and 3 ($136 \pm 29$ µmol $l^{-1}$ and $124 \pm 24$ µmol $l^{-1}$) so that, even though there was some degree of renal impairment initially, treatment did not cause any further deterioration in renal function. Serum alkaline phosphatase was unaltered by treatment. Total white cell count fell between days 0 and 3 from $10.1 \pm 1.2$ to $8.6 \pm 1.1 \times 10^9 l^{-1}$ ($P<0.05$). This was unduly influenced by two patients who received cytotoxic chemotherapy within 3 days prior to bisphosphonate treatment. Of three patients in whom total white cell count fell below the normal range ($<4 \times 10^9 l^{-1}$), this included the two who had received concomitant chemotherapy.

Three different patients received radiotherapy to their primary lesion at the same time as bisphosphonate treatment. These patients had squamous carcinoma of the lung, squamous carcinoma of the nasopharynx and transitional cell carcinoma of the bladder, and none of the three became normocalcaemic in spite of both bisphosphonate and radiotherapy. Excluding the five patients who received these concomitant therapies 73% (11/15) of patients became normocalcaemic after treatment with AHBP.

Two patients (10%) experienced pyrexia (37.5° and 38°C) within 24 h of infusion, in the apparent absence of infection. In both the temperature returned to normal by the following day. Two patients complained of nausea within 2 days of treatment, but this was non-specific and could not be conclusively attributed to the bisphosphonate. In two patients loose bowel motions were noted on the day after infusion, which may have been a related adverse effect. A third patient had severe diarrhoea that lasted for 3 days, but the patient had recurrent hypercalcaemia 6 weeks later which was retreated with aminohexane bisphosphonate without any adverse effect, and it was considered that bowel symptoms were not bisphosphonate induced.

### Discussion

This preliminary investigation demonstrates the calcium-lowering effect of aminohexane bisphosphonate in the treatment of tumour-induced hypercalcaemia. In all patients treated there was a fall in serum calcium, and 65% of patients became normocalcaemic. As with the other bisphosphonates thus far tested, this calcium-lowering activity is mediated by inhibition of bone resorption, as judged by a significant reduction in the fasting urinary excretion of calcium (Coleman & Rubens, 1988; Bonjour & Rizzoli, 1989; O'Rourke *et al.*, 1993).

The dose chosen for this study was taken from our previous experience with this bisphosphonate in the treatment of Paget's disease of bone. We observed significant decreases in disease activity with intravenous treatments of 25 or 50 mg given daily for 5 days (Atkins *et al.*, 1987). In the only previous reported use of aminohexane in hypercalcaemia, eight patients received 25–50 mg daily for 5 days, which resulted in a fall in mean serum calcium (Hamdy *et al.*, 1987; abstract, numerical results not reported). Experience with clodronate in the treatment of hypercalcaemia suggests that the use of a single infusion with an equivalent total dose to that of the 5 day treatment may be equally effective in lowering serum calcium but is more rapid in onset of effect and is obviously more convenient to administer (O'Rourke *et al.*, 1993). For this reason we decided on single-infusion therapy and opted cautiously for the lower total dose of 125 mg. It is appropriate that future studies should investigate the effects of higher and lower doses to determine whether there is a dose-response relationship.

With the dose we used serum calcium fell to normal in 65% of the patients, although there was a fall in serum calcium in all patients. As with the single infusion of clodronate, the onset of effect was rapid with a significant decrease in serum calcium by day 3. The nadir was at day 7 and the effect appeared to be maintained to 28 days.

Studies using pamidronate report a variable success rate of between 66% and 90% (Ralston *et al.*, 1988, 1989) since the response rate varies according to the mechanism of induction of hypercalcaemia (Thiebaud *et al.*, 1990). Whereas increased bone resorption is usually the major mechanism contributing to development and maintenance of tumour-induced hypercalcaemia, there is a substantial minority of patients in whom increased renal tubular reabsorption of calcium plays a significant role, probably related to the humoral secretion of PTHrP (Bonjour & Rizzoli, 1989). This is particularly the case in patients with squamous cell carcinoma or transitional cell carcinoma. In such patients, bisphosphonate therapy will inhibit bone resorption but not renal tubular reabsorption of calcium, and so there is only an incomplete response of serum calcium to treatment (Thiebaud *et al.*, 1990).

In the present study we also observed incomplete responses to treatment which may have been due to the presence of increased renal tubular reabsorption of calcium. Of the seven patients who did not become normocalcaemic following treatment, three had squamous cell carcinoma, two had transitional cell carcinoma, one had non-Hodgkin's lymphoma and one had renal cell carcinoma. The mean renal tubular reabsorption of calcium was elevated in these patients prior to treatment, and only two of the seven had documented bone metastases, which supports the suggestion that humoral mechanisms had increased renal tubular reabsorption of calcium and that this was the predominant mechanism of hypercalcaemia in these patients. Moreover, the fasting urinary calcium, an index of bone resorption, was markedly increased before treatment in those patients who showed a complete response, but was much lower in those who did not respond completely. There was no difference in initial serum calcium or creatinine between the two groups, which suggests a predominantly renal mechanism of hypercalcaemia in the patients who did not achieve normocalcaemia after treatment. Unfortunately, complete urine results from before and after treatment were available on only one of the non-responders.

With regard to acute toxicity, the dose and regimen of aminohexane bisphosphonate used in this study appeared to be less toxic than treatment with the other amino derivative, pamidronate. Hypocalcaemia did not occur and there were no reported episodes of malaise. Ten per cent of patients experienced a self-limiting mild pyrexia, with a further 10% suffering gastrointestinal symptoms. The observed fall in total white cell count after treatment was slight and not significant when those patients who had received concomitant cytotoxic chemotherapy were excluded. This compares favourably with the toxicity profile observed with the other amino bisphosphonates pamidronate and alendronate, both of which are normally associated with an acute-phase response (Mautalen *et al.*, 1984; Adami *et al.*, 1987).

We conclude that aminohexane bisphosphonate is an effective treatment for tumour-induced hypercalcaemia due to increased bone resorption, at the dose tested. It appears to have fewer side-effects than its structural analogue pamidronate. Both the duration of response and the optimum dose are not known and need to be clarified by further investigation.

The AHBP used was kindly donated by Instituto Gentilli, Italy.

### References

ADAMI, S., BHALLA, A.K., DORIZZI, R., MONTESANI, F., ROSINI, S., SALVAGNO, G. & LOCASCIO, V. (1987). The acute phase response after bisphosphonate administration. *Calcif. Tiss. Int.*, **41**, 326-331.

ATKINS, R.M., YATES, A.J.P., GRAY, R.E.S., URWIN, G.H., HAMDY, N.A.T., BENETON, M.N.C., ROSINI, S. & KANIS, J.A. (1987). Aminohexane diphosphonate in the treatment of Paget's disease of bone. *J. Bone. Min. Res.*, **2**, 273-279.

BONJOUR, J.P. & RIZZOLI, R. (1989). Pathophysiological aspects of therapeutic approaches of tumoral osteolysis and hypercalcaemia. *Rec. Res. Cancer. Res.*, **116**, 29-39.

COLEMAN, R.E. & RUBENS, R.D. (1988). 3(amino-1,1-hydroxypropylidene)bisphosphonate (APD) for hypercalcaemia of breast cancer. *Br. J. Cancer*, **56**, 621-625.

GALLACHER, S.J., RALSTON, S., PATEL, U. & BOYLE, I.T. (1989). Side-effects of pamidronate. *Lancet*, **i**, 42-43.

HAMDY, N.A.T., GRAY, R.E.S., URWIN, G.H., MURRAY, S.A., ROSINI, S. & KANIS, J.A. (1987). Effects of the diphosphonate AHDP in hypercalcaemia. In *Calcium Regulation and Bone Metabolism. Basic and Clinical Aspects*, Vol. 9. Cohn, D.V., Martin, T.J. & Meunier, P.J. (eds), p. 710. Excerpta Medica: Amsterdam.

KANIS, J.A., CUNDY, T., HEYNEN, G. & RUSSELL, R.G.G. (1980). The pathophysiology of hypercalcaemia. *Metab. Bone Dis. Rel. Res.*, **2**, 151-159.

KANIS, J.A., McCLOSKEY, E.V., O'ROURKE, N., PRESTON, E., GREAVES, M., EYRES, K. & VASIKARAN, S. (1991). Bisphosphonates in the management of hypercalcaemia of malignancy. In *Tumour-induced Hypercalcaemia and its Management*. Russell, R.G.G. & Kanis, J.A. (eds). Royal Society of Medicine: London.

MAUTALEN, C.A., CASCO, C.A., GONZALEZ, D., GHIRINGHELLI, G.R., MASSIRONI, C., FROMM, G.A. & PLANTALECH, L. (1984). Side effects of disodium aminohydroxypropylidenediphosphonate (APD) during treatment of bone diseases. *Br. Med. J.*, **288**, 828-829.

MORTON, A., DODWELL, D.J. & HOWELL, A. (1989). Disodium pamidronate for the management of hypercalcaemia of malignancy: comparative studies of single dose versus daily infusions and of infusion duration. In *Disodium Pamidronate in the Treatment of Malignancy-related Disorders*, Burckhardt (ed.), pp. 85-100. Hans Huber: Berne.

O'ROURKE, N.P., McCLOSKEY, E.V., VASIKARAN, S., EYRES, K., FERN, D. & KANIS, J.A. (1993). Effective treatment of malignant hypercalcaemia with a single infusion of clodronate. *Br. J. Cancer*, **67**, 560-563.

PERCIVAL, R.C., YATES, A.J.P., GRAY, R.E.S., GALLOWAY, J., ROGERS, K., NEAL, F.E. & KANIS, J.A. (1985). Mechanisms of malignant hypercalcaemia in carcinoma of the breast. *Br. Med. J.*, **291**, 776-779.

RALSTON, S.H., ALZAID, A.A., GALLACHER, S.J., GARDNER, M.D., COWAN, R.A. & BOYLE, I.T. (1988). Clinical experience with aminhydroxypropylidene bisphosphonate (APD) in the management of cancer-associated hypercalcaemia. *Q. J. Med.*, **68**, 825-834.

RALSTON, S.H., GALLACHER, S.J., PATEL, U., DRYBURGH, F.J., FRASER, W.D., CAVAN, R.A. & BOYLE, I.T. (1989). Comparison of three intravenous bisphosphonates in cancer associated hypercalcaemia. *Lancet*, **i**, 1180-1182.

RIZZOLI, R., BUCHS, B. & BONJOUR, J.P. (1992). Effect of a single infusion of alendronate in malignant hypercalcaemia: dose dependency and comparison with clodronate. *Int. J. Cancer*, **50**, 706–712.

SHINODA, H., ADAMEK, G., FELIX, R., FLEISCH, H., SCHENK, R. & HAGAN, P. (1983). Structure–activity relationship of various bisphosphonates. *Calcif. Tiss. Int.*, **35**, 87–99.

THIEBAUD, D., JAEGER, P. & BURCKHARDT, P. (1990). Response to treatment of malignant hypercalcaemia with bisphosphonate AHPrBP (APD): respective role of kidney and bone. *J. Bone Min. Res.*, **5**, 221–226.

URWIN, G.H., YATES, A.J.P., GRAY, R.E.S., HAMDY, N.A.T., McCLOSKEY, E.V., PRESTON, E. & KANIS, J.A. (1987). Treatment of hypercalcaemia of malignancy with intravenous clodronate. *Bone*, **8** (Suppl. 1), 43–51.

VAN HOLTEN-VERZANTVOORT, A.T., BIJVOET, O.L.M., CLETON, F.J., HERMANS, J. & KROON, D.M. (1987). Reduced morbidity from skeletal metastases in breast cancer patients during long-term bisphosphonate (APD) treatment. *Lancet*, **i**, 983–985.